# Mechanism of Labor

**Published:** 1887-10

**Authors:** T. Lazarewitch

**Affiliations:** St. Petersburg, Russia


### *THE MECHANISM OF LABOR AND THE NORMAL FORCEPS.*

By Professor T. LAZAREWITCH, M. D., of St. Petersburg, Russia.

After calling attention to the factors concerned in the mechanism of labor, and the necessity of an accurate knowledge of the mechanics of the process, he described a forceps which he had devised, having straight parallel blades, and locking with a single tenon and screw.

His conclusions were:

1. That forceps be considered as a continuation of the hand as feelers.

2. That the less the dimensions of the blades the better they could be guided.

3. That detrimental action increases with the size of the blades.

4. Convex margins should not be so thin as to cut, or so thick as to obstruct.

5. That the instrument should lock easily, but allow slight longitudinal rotation of the blades.

6. Blades should be parallel.

7. Handles designed for convenience in guiding and the avoidance of injurious pressure.

8. Should be of smooth metal, so as to be easily made aseptic.

9. That the pelvic curve was injudicious, detrimental and difficult to employ.

10. That his parallel normal forceps filled all these conditions.

Dr. Opie thought that most forceps had merit in proportion to the skill and familiarity in their use by the individual operator. It was not so much the instrument as the man. We should not try to do by mechanism what the skillful hand may execute. A properly-educated touch and hand were the best means of warding off dangers incident to the use of the forceps. He believed in the use of a moderate pelvic curve.

